# Combining HPV DNA load with p16/Ki-67 staining to detect cervical precancerous lesions and predict the progression of CIN1–2 lesions

**DOI:** 10.1186/s12985-019-1225-6

**Published:** 2019-10-16

**Authors:** Yuejie Li, Jie Liu, Li Gong, Xingwang Sun, Wenbo Long

**Affiliations:** Pathology Department of the First Affiliated Hospital, Southwest Medical University, Taiping Street in Sichuan province Luzhou City Jiangyang District No. 25, Sichuan, China

**Keywords:** HPV DNA load, p16/Ki-67/HPV DNA load co-test, Diagnosis and prognosis, Immunohistochemistry

## Abstract

**Background:**

Human Papilloma Virus (HPV) DNA tests are highly sensitive and can triage women with mild lesions, improving the prognosis and diagnosis of cervical lesions. However, additional efficient strategies should be developed to improve the specificity of these tests.

**Methods:**

This study aimed to evaluate the clinical value of HPV DNA load in improving the diagnosis and prognosis of cervical lesions by p16/Ki-67 testing. Histological samples were collected from 350 women with HR-HPV genotyping and analyzed by qRT-PCR. Immunohistochemical staining was used to assess p16 and Ki-67 expression and clinical performance characteristics were calculated.

**Results:**

Of the cases, 271 had detectable HR-HPV infection, in which HPV-16 was most prevalent (52.0%), followed by HPV-58 (22.5%). P16/Ki-67-positivity increased with histological severity but not for HR-HPV infection. Amongst the 13 HR-HPV genotypes, only HPV-16 (*P* = 0.016) and HPV-58 (*P* = 0.004) viral loads significantly correlated with lesion severity. The P16/Ki-67/HPV DNA load co-test indicated an increased sensitivity for the detection of cervical intraepithelial neoplasia (CIN) lesions compared to p16/Ki-67 staining in HPV-16 and/or 58 positive cases. Viral load did not improve the sensitivity of p16/Ki-67 co-test in non-HPV-16 or 58 positive cases. The clinical performance of the p16/Ki-67/HPV DNA load co-test was limited for the prediction of the outcome of CIN1 lesions. However, amongst the 12 HPV-16 and/or 58 positive CIN2 cases in which return visit results were obtained, the behavior of the lesions could be predicted, with a sensitivity, specificity, positive prediction rate (PPV), and negative prediction rate (NPV) of 0.667, 1, 1 and 0.5, respectively.

**Conclusion:**

Combination of the assessment of HPV DNA load with the intensity of p16 and Ki-67 staining could increase the sensitivity of CIN lesion diagnosis and predict the outcome of CIN2 in patients with a HPV-16 and/or 58 infection.

## Introduction

Human papillomavirus (HPV) is a major cause of cervical cancer (CCa) and its screening can improve the diagnosis of both CCa and precancerous lesions [1, 2]. HPV DNA tests are highly sensitive and can triage women with low-grade or equivocal cytological abnormalities, improving prognosis assessments, and increasing the diagnostic accuracy of cervical intraepithelial neoplasia (CIN) lesions [3]. However, the specificity of HPV assessments for precancerous CCa is poor as the majority of HPV infections are naturally cleared [4]. Methods to enhance the diagnostic accuracy of these tests in the clinic, are therefore highly sought. More specific molecular markers of CCa have been identified based on studies of HPV-induced carcinogenesis. Host gene expression is influenced by the expression of oncogenic HPV proteins including those involved in cell cycle regulation, including p16 and Ki-67.

P16 is a tumor suppressor that inhibits cyclin-dependent kinases (CDK) 4 and 6, which regulate the checkpoint of the G1 to the S phase. Overexpression of p16 is associated with functional inactivation of the retinoblastoma (Rb) protein that is induced by the deregulation of HPV E6 and E7 proteins [5]. Immunohistochemical staining of p16 has been widely evaluated as a CCa biomarker as an overwhelming majority of High-risk HPV (HR-HPV) positive cases with severe CIN lesions display high p16 expression [6]. However, some normal cervical tissues express p16 positively and a small fraction of CIN2 or worse cases produce weak or negative p16 staining [7], therefore limiting the use of p16 immunostaining for the detection of cervical lesions. Nuclear-located protein Ki-67 is a proliferation marker that expresses in G1, S, G2, and M stages of the cell cycle. High expression of Ki-67 has been associated with the severity of cervical lesions but not with HPV infection [8]. Thus, Ki-67 combined with p16 staining has been proposed as auxiliary test for the diagnosis of high-risk precursor or CCa lesions to improve the efficiency of CCa detection [9].

Recently, p16/Ki-67 staining has been proposed as a primary screening strategy to triage women with atypical cells of undetermined significance (ASCUS) and Low-grade squamous intraepithelial lesion (LSIL). The use of p16/Ki-67 as CCa biomarkers yields high sensitivity for CIN2+ and CIN3+ and decreases the inter-observer variability of cytology scoring [10–12]. The specificity of p16/Ki-67 dual-staining is however limited, as it fails to identify some CIN lesions. In this study, we analyzed the performance of the p16/Ki-67 test combined with the assessment of the HPV DNA load in cervical paraffin specimens to detect CIN+ lesions. We focused on the combined ability of the HPV DNA load/p16/Ki-67 co-test to identify women with CIN1 and CIN2 lesions.

## Methods

### Study population and specimen collection

Specimens were collected from women who underwent biopsy from January 2016 to June 2018 in the First Affiliated Hospital of Southwest Medical University. Over 600 specimens were collected during the two-year period, of which 50% of samples were excluded. Exclusion criteria encompassed previous operation for cervical disease (including the loop electrosurgical excision procedure (LEEP), cold-knife conization, hysterectomy, laser therapy, chemotherapy/radiation treatment, pregnancy, and malignant diseases outside the cervix. A total of 350 eligible specimens were collected from women ranging from 20 to 72 years with an average age of 43.49 ± 9.56. Prior to biopsy, cervical cells and fragments were obtained from the internal genitalia with a cytobrush. Smears were collected for DNA extraction. Biopsy specimens were fixed in formalin, embedded in paraffin, and stained with hematoxylin-eosin. Sections were used for subsequent p16 and Ki-67 immunohistochemical analysis.

### HPV DNA genotyping and quantification

Liquid-based cytological samples (50 μL) were pelleted and 200 μL of denaturing reagent was added to the pellet. Tubes were incubated at 100 °C for 10 min, followed by centrifugation. Supernatants were collected and the nucleic acid concentration measured using a NanoDrop 2000 spectrophotometer. HPV DNA was amplified via qRT-PCR and screened using the Slan-96P Real time PCR System (Hongshi medical technology Co. Ltd. Shanghai, China). The following HR-HPV genotypes were assayed: HPV-16, 18, 31, 33, 35, 39, 45, 51, 52, 56, 58, 59, and 68. For HPV DNA quantification assays, the levels of the dual specificity tyrosine phosphorylation regulated kinase 1A (DYRK1a) was employed as reference. Viral loads were calculated according to the formula: viral load = 2 ^ (reference CT – objective CT). CT: cycle threshold.

### Histological diagnosis

In this study, cytological samples and biopsies were taken simultaneously. Histological results were collected from the records of pathologists. Results were interpreted by two experts and if an agreement was not obtained, a third expert was used to finalize the diagnosis. When multiple results were recorded in single patients, the most severe histological diagnosis was used for analysis. A consensus diagnosis was established on most available cervical specimens. Histological diagnoses were performed according to the WHO 2014 classification criteria and were classified as cervicitis (including cervical hypertrophy, nabothian cysts, erosion, bleeding, hyperplasia and polyp), CIN1 (LSIL), CIN2, and CIN3 (high grade squamous intraepithelial lesion, HSIL), and invasive CCa. To investigate the differences between CIN2 and CIN3, we did not abandon the CIN terminology and classified intraepithelial lesions into CIN1, CIN2, and CIN3.

### Immunohistochemistry staining and scoring

Serial sections (4 μm) from formalin-fixed paraffin embedded blocks were sliced and mounted onto glass slides. After sequential deparaffinization and rehydration, antigen retrieval was performed in a water bath following heating in a microwave. Slides were treated with 0.3% H2O2 for 15 mins to quench endogenous peroxidase. After blocking in 3% goat serum, slides were labeled with primary antibodies overnight at 4 °C. A secondary antibody reagent was employed for visualization after washing. Slides were incubated with DAB (3,3′-diaminobenzidine). Substrate chromogen solution and counterstaining was performed with Mayer’s hematoxylin and slides were mounted. Negative control slides lacking primary antibodies were included for each staining protocol. The recommendations of WHO considered a positive p16 staining as a case with a strong diffuse staining in at least one third of the thickness of the epithelium, starting from the bottom. For quantitative analysis of both p16 and Ki-67 immunostaining, we scored immunohistochemical results according to the description by Koo et al. [13]. The percentage of positive cells was determined at × 400 magnification and assigned to one of five of categories: (a) 0: < 5%; (b) 1: 5–25%; (c) 2: 25–50%; (d) 3: 50–75%; and (e) 4: > 75%. The intensity of immunostaining was scored as: (a) 1: weak; (b) 2: moderate; and (c) 3: intense. If no areas of heterogeneous staining were observed, the predominant area was considered for further investigation. Positive cell percentage scores and staining intensity were multiplied to produce a weighted score for each case. For defining positive events of p16 and Ki-67 staining, the threshold for differentiating between positive and negative staining was set at 2 and 1 for interpretation of p16 and Ki-67 cases, respectively. In this study, the optimal cut-off values of p16 and Ki-67 were determined by the Receiver-Operator-Curve (ROC) analysis. A score of 2 or greater was considered positive for p16 expression, whereas a score of 1 or greater was considered positive for Ki-67.

### Statistical analysis

Descriptive statistics were employed for data characterization. A Pearson’s χ2 test was used to evaluate significant differences between the designated groups. A Pearson’s correlation analysis was performed to analyze the relationship between HPV DNA load, the severity of intraepithelial neoplasms, and p16/Ki-67 staining scores. During the analysis, CIN1, CIN2, and CIN3 cases were scored as 1, 2, and 3, respectively, and CCa was scored as 4. Those lacking intraepithelial lesions were scored as 0. Sensitivity, specificity, positive predictive value (PPV), negative predictive value (NPV) with 95%-confidence intervals were calculated for two different endpoints. Sensitivity and specificity of the confidence intervals were plotted on a ROC characteristics graph using MedCalc software. For p16 and Ki-67 tests, the ROC curve was plotted by using the p16 score or Ki-67 score. However, for Ki-67/p16, HPV load/Ki-67, HPV load/p16, and HPV load/Ki-67/p16 test, the logistic curve was calculated first, then the predicted values on the curve were adopted to generate ROC curve.

## Results

### Histology and HPV prevalence

A total of 350 women were included in the analysis. The median age was 43.5 (± 9.56) years (complete range: 20–72). Of the 271 women with available HR-HPV results, 141 (52.0%) were positive for HPV-16, 61 (22.5%) were positive for HPV-58, and 56 (20.7%) were positive for HPV-52 (Table 1 and Additional file 1: Table S1). Amongst the 350 women, 84 (24%) had cervicitis, 77 (22%) had CIN1, 68 (19.4%) had CIN2, 89 (25.4%) had CIN3 and 32 (9.1%) had CCa. There was an increasing proportion of HR-HPV with increasing histological severity, from 58.3% in cervicitis, 64.9% in CIN1, 82.4% in CIN2, 97.8% in CIN3, to 90.6% in CCa.

**Table 1:** Distribution of HPV genotypes according to histopathological diagnosis

Type	Cervicitis	CIN1	CIN2	CIN3	cancer	Total	χ^2^ value	*P* value
No HPV	35	27	12	2	3	79	/	/
HPV-16	14	17	24	61	25	141	76.408^**^	< 0.001
HPV-18	9	1	3	1	2	16	6.535	0.258
HPV-31	3	5	2	6	0	16	22.199^**^	< 0.001
HPV-33	4	2	7	7	1	21	26.592^**^	< 0.001
HPV-35	5	1	4	2	1	13	8.849	0.115
HPV-39	6	4	1	1	1	13	1.792	0.877
HPV-45	1	0	1	1	0	3	9.314	0.097
HPV-51	5	2	5	4	0	16	15.747^**^	0.008
HPV-52	15	17	12	10	2	56	12.243^*^	0.032
HPV-56	4	4	1	1	0	10	2.136	0.83
HPV-58	9	15	17	19	1	61	32.6^**^	< 0.001
HPV-59	2	1	2	3	0	8	17.891^**^	0.003
HPV-68	1	4	3	2	1	11	9.782	0.082
Total	84	77	68	89	32	350	32.329^**^	< 0.001

* at the *P* < 0.05 level

** at the *P* < 0.01 level

### Assessment of p16, Ki-67 and p16/Ki-67 positivity by histological analysis

We stratified women by HPV status into three age categories: (1) < 30; (2) ≥30 and < 45; and (3) ≥45. In total, an increasing proportion of p16/Ki-67 positive subjects with increasing histological severity were observed. These ranged from 9.5% in women with cervicitis, 22.1% in women with CIN1, 73.5% in women with CIN2, 88.8% in women with CIN3, to 96.9% in women with CCa (Table 2). The proportion of Ki-67 positive subjects was higher than the p16 positive subjects in women with or without neoplasia. HPV-positive women had a higher percentage of p16, Ki-67 and p16/Ki-67 positivity compared to HPV-negative women, which was stratified by age. However, in women with cervicitis and CIN1, those who were HPV-positive had a higher percentage of Ki-67 positivity only in the ≥30 and < 45 age category. In general, the intensity of p16 and Ki-67 staining increased with the severity of the lesions. Only a select number of cases in women with CIN1 had a high intensity of p16/Ki-67 staining.

**Table 2:** P16 and Ki-67 positivity in histology categories stratified by age and HPV infection

Category	Cervicitis	CIN1	CIN2	CIN3	cancer	Total
HPV+ / < 30	4	6	3	4	0	17
% (p16+, Ki-67+, D+)	0, 25, 0	50, 66.7, 16.7	100, 100, 100	75, 100, 75	N/A	52.9, 70.6, 41.2
HPV- / < 30	2	3	1	0	0	6
% (p16+, Ki-67+, D+)	0, 50, 0	0, 66.7, 0	100, 100, 100	N/A	N/A	16.7, 66.7, 16.7
HPV+ / ≥30, < 45	18	28	27	51	9	133
% (p16+, Ki-67+, D+)	11.1, 66.7, 11.1	35.7, 60.7, 25	81.5, 85.2, 70.4	86.3, 96.1, 84.3	100, 100, 100	64.7, 82.7, 59.4
HPV- / ≥30, < 45	13	13	9	1	1	37
% (p16+, Ki-67+, D+)	0, 38.5, 0	0, 69.2, 0	76.9, 88.9, 76.9	100, 100, 100	100, 100, 100	27, 70.3, 27
HPV+ /≥45	27	16	26	32	20	121
% (p16+, Ki-67+, D+)	11.1, 40.7, 7.4	37.5, 87.5, 37.5	80.8, 88.5, 73.1	96.9, 100, 96.9	95, 100, 95	66.1, 82.6, 63.6
HPV- / ≥45	20	11	2	1	2	36
% (p16+, Ki-67+, D+)	20, 55, 20	27.3, 90.9, 27.3	50, 100, 50	100, 100, 100	100, 100, 100	30.6, 72.2, 30.6
Total	84	77	68	89	32	350
% (p16+, Ki-67+, D+)	10.7 48.8, 9.5	28.6, 72.7, 22.1	80.9, 88.2, 73.5	89.9, 97.8, 88.8	96.9, 100, 96.9	56.3, 78.9, 52.9

### Correlation analysis between HPV DNA load and lesion severity, the p16 score, and the Ki-67 score

The total HPV DNA load significantly correlated with p16 (*P* = 0.015) immunohistochemistry staining, but did not significantly correlate with lesion severity or Ki-67 staining scores (Table 3). Regarding specific HPV genotypes, only HPV-16 (*P* = 0.016) and HPV-58 (*P* = 0.004) loads significantly correlated with lesion severity. The HPV-16 load also significantly correlated with the p16 staining score (*P* = 0.047), but did not correlate with Ki-67 staining. The HPV DNA load of any subtype did not significantly correlate with Ki-67 staining score, though a strong correlation between the p16 and Ki-67 scores were noted (*P* < 0.001).

**Table 3:** Correlation analysis between HPV DNA load and lesion severity, p16 score, Ki-67 score

Type	Lesion severity (P value)	p16 score (P value)	Ki-67 score (P value)
HPV-16	0.203^*^ (0.016)	0.168^*^ (0.047)	0.035 (0.679)
HPV-18	−0.168 (0.533)	−0.151 (0.578)	−0.143 (0.598)
HPV-31	0.280 (0.293)	0.478 (0.061)	0.433 (0.094)
HPV-33	−0.002 (0.994)	0.367 (0.102)	−0.272 (0.232)
HPV-35	−0.437 (0.179)	− 0.369 (0.264)	− 0.491 (0.125)
HPV-39	−0.108 (0.724)	− 0.262 (0.388)	−0.21 (0.491)
HPV-45	0.332 (0.784)	0.704 (0.503)	0.869 (0.33)
HPV-51	−0.257 (0.336)	−0.295 (0.268)	− 0.087 (0.749)
HPV-52	0.012 (0.931)	−0.016 (0.907)	0.101 (0.457)
HPV-56	0.031 (0.932)	−0.021 (0.955)	−0.012 (0.975)
HPV-58	0.364^**^ (0.004)	0.180 (0.165)	0.106 (0.416)
HPV-59	−0.241 (0.566)	−0.291 (0.485)	− 0.171 (0.686)
HPV-68	−0.437 (0.179)	− 0.369 (0.264)	−0.491 (0.125)
Total	0.111 (0.068)	0.147^*^ (0.015)	0.062 (0.307)

* at the P < 0.05 level

** at the P < 0.01 level

### Clinical performance of HPV load, p16, and Ki-67 testing in the study population

**Fig. 1:**
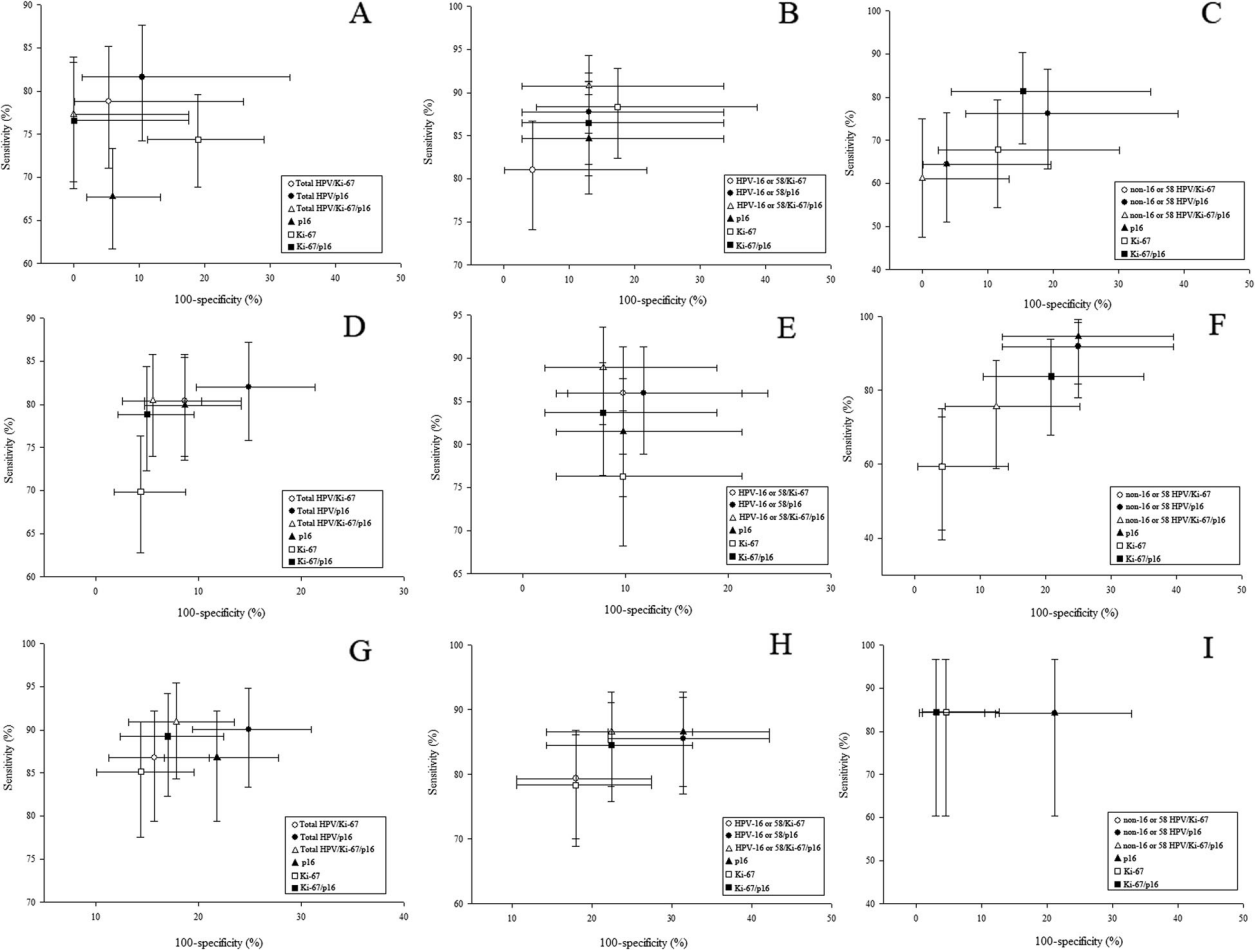
Sensitivity and specificity of the p16/Ki-67/HPV load co-test to detect CIN in a histopathologically diagnosed population. (**a**, **d** & **g**) The assessment of 13 genotypes of HR-HPV DNA load were combined with p16 and Ki-67 tests to detect CIN1+, CIN2+ and CIN3+ lesions in the 350 cases, respectively; (**b**, **e**, & **h**) HPV-16 and/or 58 DNA load assessments were combined with p16 and Ki-67 tests to detect CIN1+, CIN2+ and CIN3+ lesions in the 186 cases with HPV-16 and/or 58 infection, respectively; (**c**, **f**, & **i**) Non-16 or 58-HPV DNA load assessments were combined with the p16 and Ki-67 tests to detect CIN1+, CIN2+ and CIN3+ lesions in the 85 cases with non-16 or 58 HPVs infection, respectively

We analyzed the clinical performance characteristics of the HPV load, p16 and Ki-67, to detect CIN amongst the study population, stratified by HPV-16 and/or 58 infection (Fig. 1). Overall, the combined p16 and Ki-67 results used to detect CIN1+, CIN2+ and CIN3+ lesions showed higher sensitivity and specificity than those of p16 or Ki-67 individually. The clinical performance of the total HPV load/p16/Ki-67 co-test to detect lesions in the 350 cases did not outperform p16/Ki-67 co-testing. However, amongst the 186 women with HPV-16 and/or 58 infection, the sensitivity of the HPV-16 and/or 58 load/p16/Ki-67 co-test was higher for CIN and more severe lesions when compared to p16/Ki-67 co-testing alone. The sensitivity (CI 95%) was 0.905 (0.853–0.943), 0.889 (0.823–0.936) and 0.866 (0.782–0.927) for the detection of CIN1+, CIN2+ and CIN3+, respectively. Comparatively, the combined DNA load of non-16 or 58-HPVs with p16 and Ki-67 staining showed no improved clinical performance for the detection of CIN lesions, or lesions of greater severity.

### Clinical analysis of the HPV DNA load, p16 and Ki-67 testing for stratifying cases with HPV infection

We estimated the performance of the HPV DNA load in combination with p16/Ki-67 staining to stratify women with CIN lesions. For HPV-16 and/or 58 positive women, the combined HPV DNA load/p16/Ki-67 co-test most efficiently detected the lesions. The sensitivity and specificity were 0.908 and 0.87 for CIN1+, 0.889 and 0.922 for CIN2+ and 0.866 and 0.775 for CIN3+, respectively (Table 4). HPV/p16/Ki-67 co-test positivity was 81.7, 67.2 and 57% for the detection of CIN1+, CIN2+ and CIN3+, respectively. For non-16 or 58-HPV positive women, the most efficient method to detect the lesions was p16/Ki-67 co-testing for CIN1+ and CIN3+, and p16 staining for CIN2+. Positivity for CIN1+ and CIN3+ were 62.4 and 22.4%, respectively. Positivity for CIN2+ was 51.8%. Ki-67 had a poor sensitivity (0.595) for the detection of CIN2+ amongst non-16 or 58-HPV positive women, which greatly reduced the performance of p16/Ki-67 co-assessments. For HPV negative women, the p16/Ki-67 co-test most efficiently detected CIN and more severe lesions. Whilst the sensitivity was high, the PPV values were as low as 0.699, 0.696 and 0.417 for the detection of CIN1+, CIN2+ and CIN3+, respectively.

**Table 4:** Clinical performance of the most efficient test to detect CIN1+, CIN2+ and CIN3+ lesions

	HPV infection	Test	Sensitivity (%)	Specificity (%)	PPV (%)	NPV (%)
CINI+	no	p16/Ki-67	88.64 (75.4–96.2)	51.43 (34–68.6)	69.9 (64.6–75)	78.2 (68.5–85.7)
	HPV-16 and/or 58	p16/Ki-67/HPV load	90.8 (85.3–94.3)	86.96 (66.4–97.2)	98 (95.1–99.3)	57.1 (47.8–62.5)
	others	p16/Ki-67	81.36 (69.1–90.3)	84.63 (65.1–95.6)	92.3 (85.5–97.6)	66.67 (58.1–73.9)
CINII+	no	p16/Ki-67	94.12 (71.3–99.9)	88.71 (78.1–95.3)	69.6 (54.8–80)	98.2 (92.2–100)
	HPV-16 and/or 58	p16/Ki-67/HPV load	88.89 (82.3–93.6)	92.16 (81.1–97.8)	95.8 (92.6–99.1)	75.8 (67.6–82)
	others	p16	94.59 (81.8–99.3)	75 (60.4–86.4)	74.5 (65.9–82.3)	94.7 (86–99)
CINIII+	no	p16/Ki-67	100 (47.8–100)	90.54 (81.5–96.1)	41.7 (26.7–44.4)	100 (96.5–100)
	HPV-16 and/or 58	p16/Ki-67/HPV load	86.6 (78.2–92.7)	77.53 (67.4–85.7)	80.8 (75.6–85.4)	84.1 (78.4–89.6)
	others	p16/Ki-67	84.21 (60.4–96.6)	96.97 (89.5–99.6)	88.9 (72.4–97.5)	95.5 (89.8–99)

### Performance of the p16/Ki-67/HPV co-test to predict the progression of CIN1 and CIN2

The behaviors of CIN1 and CIN2 lesions were predicted by p16, p16/Ki-67 or p16/Ki-67/HPV co-tests (Table 4). Despite 145 patients possessing CIN1 or CIN2 lesions, only 21 cases of CIN1 and 27 cases of CIN2 were used for predictions, as these cases had return visits and did not undergo surgery (Additional file 2: Table S2). Positive predictions were recorded when the lesions did not regress, and negative means were recorded. The coincidence rates between the prediction results and return visits were analyzed (Table 5). The combination of HPV DNA load with p16/Ki-67 staining effectively predicted the behavior of the 12 CIN2 cases of HPV-16 and/or 58 infection, in which the sensitivity, specificity, PPV and NPV were 0.667, 1, 1 and 0.5, respectively. However, the inclusion of the HPV DNA load test did not improve the efficiency of p16/Ki-67 co-test for the prediction of CIN1 lesions, with the sensitivity, specificity, PPV and NPV for the 15 cases being only 0.333, 0.667, 0.4 and 0.6.

**Table 5:** The performance of p16, p16/Ki-67 and p16/Ki-67/HPV test to predict the behavior of CIN1 and CIN2 lesions

Initial diagnosis	Number	Interval month	Return visit result			Prediction result		Coincidence rate (%)			
			Progression	Persistence	Regression	Positivity	Negativity	No HPV infection	HPV-16 and/or 58 infection	Other HPVs infection	Total
CIN1	21	4.3 ± 3.37	3	5	13	11	10	1/1 (100)	8/15 (53.3)	3/5 (60)	12/21 (57.1)
CIN2	27	3.02 ± 1.44	6	6	15	21	6	1/4 (25)	9/12 (75)	6/11 (54.5)	16/27 (59.3)
Total	48	3.58 ± 2.53	9	11	28	32	16	2/5 (40)	17/27 (63)	8/16 (50)	27/48 (56.3)

## Discussion

HPV screening is recognized as a necessary but insufficient factor during CCa development. Whilst HPV DNA tests are highly sensitive for the detection of precancerous lesions, they lack the ability to differentiate precancerous cases from HPV infected individuals [14]. The coexpression of p16 and Ki-67 was developed as an auxiliary marker of cervical precancers [11, 12], but a series of studies reported that an increased risk of high-grade CIN or CCa is associated with high HPV DNA loads [15, 16], suggesting that HPV is a marker to predict cervical neoplasia. In previous studies, the combined assessment of p16/Ki-67/HPV was employed to detect CIN. We evaluated the clinical performance of the p16/Ki-67 co-test independently, and in combination with HPV DNA loads, to detect cervical precancerous lesions. Patients were stratified into three categories according to HPV genotype, including HPV negative cases, HPV-16 and/or 58 positive cases, and non-16 or 58-HPV positive cases. HPV-16 and/or 58 positive cases instead of HPV-18 cases were separated because HPV-18 is of low prevalent in southwest China and its viral load is not associated with the severity of lesions [9–11, 17, 18].

Following stratification by age and HPV status, p16/Ki-67 positivity increased with the severity of CIN and worse lesions, in accordance with previous reports [11, 19]. Compared to women aged less than 30 years, HR-HPV positive women ≥30 years had higher rates of CIN2+ cases. Based on the documented risk of the CIN2+ association with persistent lesions and the high rate of severe lesions with HR-HPV infections [12, 16, 20], it was assumed that CIN2+ is related to HR-HPV amongst women ≥30 years who possess the highest risk of cancer invasion.

Though persistent infections are required for the conversion of low-grade to high-grade lesions or cancer, the clearance of HR-HPV infections rarely occurs in patients with high viral loads [21]. As such, the HPV viral load may act as a marker to detect cervical lesions and can contribute to the triage of afflicted patients. In this study, we calculated the viral loads of HR-HPVs and combined them with p16/Ki-67 assessments. It was interesting to note that the combination of all 13 HPV loads showed no improvement in either the sensitivity or specificity of the p16/Ki-67 test for the prediction of CIN or more severe lesions. However, amongst those patients with HPV-16 and/or 58 infection, a combination of the HPV-16 and/or 58 DNA load with p16/Ki-67 staining increased the sensitivity of the detection of CIN1+ and CIN2+ lesions, though p16 and Ki-67 tests were proven to be a highly sensitive method to estimate CIN2+ cytological cases [9, 22, 23]. Whilst the combination of HPV-16 and/or 58 DNA loads achieved only a modest improvement in the detection of CIN3+, the elevated expression of p16/Ki-67 closely correlated with severe neoplastic lesions or more severe HPV positive cases [9, 23, 24]. In comparison to the sensitivity, the specificity of the detection of neoplastic lesions was not improved the inclusion of HPV DNA loads. Surprisingly, amongst patients with non-16 or 58-HPV infections, p16/Ki-67/HPV co-test showed a higher specificity than p16/Ki-67 co-test for the detection of both CIN1+ and CIN2+, though the sensitivity of the test significantly declined. It is therefore necessary to discriminate HPV-16 and/or 58 cases and non-16 or 58-HPV infected cases if the HPV DNA load is applied as an auxiliary method to triage HPV infected patients.

Though CIN1 lesions with p16 staining had a higher tendency to progress to high grade lesions [25], it was difficult to predict the progression or regression of CIN1 [26]. Only a small area of the CIN1 lesions progressed to CIN2 or worse, and 15–30% of CIN2 or more severe lesions undergo regression [27, 28]. Thus, some CIN lesions that were referred for surgical management may be over-treated and naturally regress [29]. This suggests that the development of a method to predict the progression of CIN is of high clinical value. We found that the combination of HPV DNA load and p16/Ki-67 staining could effectively predict the outcome of CIN2 lesions in patients infected with HPV-16 and/or 58. The positivity was 75% and the specificity was 1, indicating that the referral to treatment or operation would be reduced by 25% if p16/Ki-67/HPV load co-testing was used as an additional triage assessment in this population. However, both the p16/Ki-67/HPV load and p16/Ki-67 co-tests showed limited effects in the prediction of the progression of CIN1 lesions, though the presence of HPV DNA and p16 and Ki-67 staining were necessary to distinguish high-risk oncogenic cases from those of low-risk [19, 25, 30]. The prediction of either CIN1 progression or regression is challenging and can be influenced by the HPV genotype, genomic mutations, and host immune responses [4, 25]. We demonstrated that the assessment of the HPV load could improve the sensitivity of the p16/Ki-67 method for the diagnosis of CIN1 cases, but could not improve the prediction of the outcome of the lesions. Although biomarkers have been developed to manage the stratification of CIN1 patients [31–33], we demonstrated that P16/Ki-67 co-test was the most efficient method to accurately define CIN1 prognosis in patients with and without HPV-16 infection [19, 25, 34]. Unlike those infected with HPV-16 and/or 58, it was difficult to predict the behavior of CIN2 lesions amongst non-16 or 58-HPV infected patients. We speculate that this is due to the genetic differences that exist amongst HPV subtypes [35], leading to diverse mechanisms of HPV-induced precancerous lesions or lesions of heightened severity.

In this study, we determined that the p16/Ki-67/HPV load co-test may have higher clinical value than the p16/Ki-67 co-test for detecting lesions in HPV-16 and/or 58 positive cases. However, the effect of the combination was not very strong, especially it was hard to improve the specificity of detection in those cases. We estimated the main cause may be that the genome variation and gene expression deregulation in HR-HPV infected tissues are very complex. Even for the same HR-HPV, the DNA load of the HR-HPV is not simply a linear function with genomic and expression abnormalities. Therefore, additional factors and assays should be considered to improve the combination if the combination could be employed as an alternative method to triage patients with HR-HPV infection.

## Conclusions

In a population with clear disease ascertainment due to biopsy and histological diagnosis, we demonstrate that the combined p16/Ki-67/HPV load co-test has a higher sensitivity than p16/Ki-67 co-test for the detection of cervical precancers in HPV-16 and/or 58 positive cases. We further show that the P16/Ki-67/HPV load co-test is an effective method to predict the progression of CIN2 lesions in these cases. Further studies are now required to evaluate the potential role of the P16/Ki-67/HPV load co-test as a triage marker, and larger numbers of CIN cases are required to identify optimal cut off points to effectively triage CCa patients.

## Supplementary information

**Additional file 1: Table S1.** Dignoses, qRT-PCR amplifications and immunostaining analyses of the 350 cases.

**Additional file 2: Table S2.** The data of the p16/Ki-67/HPV co-test to predict the progression of CIN1 and CIN2 for the 48 cases.

## Data Availability

All data generated or analyzed during this study are included in this manuscript [and its supplementary information files].

## Abbreviations

ASCUS: Atypical cells of undetermined significance
CCa: Cervical cancer
CDK: Cyclin-dependent kinases
CIN: Cervical intraepithelial neoplasia
HPV: Human papilloma virus
HR-HPV: High-risk HPV
HSIL: High-grade squamous intraepithelial lesion
LEEP: Loop electrosurgical excision procedure
LSIL: Low-grade squamous intraepithelial lesion
NPV: Negative predictive value
PPV: Positive predictive value
Rb: Retinoblastoma
ROC: Receiver-operator-curve
